# Acute Myelogenous Leukemia With Trisomy 8 and Concomitant Acquired Factor VII Deficiency

**DOI:** 10.1177/2324709619872657

**Published:** 2019-09-09

**Authors:** Leila Moosavi, Jonathan Bowen, Jeffrey Coleman, Arash Heidari, Everardo Cobos

**Affiliations:** 1 Kern Medical Center—UCLA, Bakersfield, CA, USA; 2 Ross University, Miramar, FL, USA

**Keywords:** acute myelogenous leukemia, trisomy 8, factor VII deficiency, cytogenetic abnormality, vitamin K

## Abstract

Acquired isolated factor VII deficiency is a rare bleeding disorder and has been reported in 31 cases. This is in contrast to congenital factor VII deficiency, which while also infrequent is the most common rare congenital bleeding disorder. Acquired isolated factor VII deficiency has been described primarily in patients with solid malignancies, sepsis, and in the presence of anti-factor VII autoantibodies. We report a case of acute myelogenous leukemia with an associated trisomy 8 cytogenetic abnormality presenting with factor VII deficiency. The factor VII deficiency cleared after induction chemotherapy and with the disappearance of the cytogenetic and molecular abnormalities. We discuss a possible link between trisomy 8 and vitamin K metabolism, which might result in acquired factor VII deficiency in acute myelogenous leukemia.

## Introduction

Acute myeloid leukemia (AML) is a heterogeneous disorder that results from a block in the differentiation of hematopoietic progenitor cells along with uncontrolled proliferation. Trisomy 8 (+8) is one of the most common numerical chromosome abnormalities reported in AML, with the occurrence of 9% of adult patients, classified as intermediate prognosis.^1^ Acquired factor VII deficiency has been reported in 31 cases and has been described associated with malignancy, infections, sepsis, postoperatively, aplastic anemia, amyloidosis, autoantibodies to factor VII, and in association with bone marrow transplantation.^2-8^ There are only 2 prior reported cases of acquired factor VII deficiency in association with AML described in the literature.^2,3^ We report the third case of AML associated with acquired factor VII deficiency and the second case of AML associated with trisomy 8 cytogenetic abnormality.

## Case Report

A 41-year-old Hispanic female with no significant past medical history presented to the emergency department with a 4-week history of fatigue, malaise, spontaneous ecchymosis, bleeding gums, and subjective fevers. The patient did not have any personal or family history involving autoimmune, hematological, or oncological pathologies. Physical examination was significant for bilateral upper extremity ecchymosis, pale conjunctivae, mucosal pallor, and scattered petechiae throughout her body. Initial laboratory tests revealed severe pancytopenia and circulating blasts suggestive of underlying leukemia (Table 1). Initial coagulation panel on admission was significant for slightly elevated prothrombin time (PT) at 15.2 seconds, while international normalized ratio (INR) and partial thromboplastin time were within normal limits (Table 2). The new-onset and severe pancytopenia, circulating blasts on peripheral smear on presentation prompted us to pursue with bone marrow biopsy to investigate for underlying malignancy.

**Table 1.** Complete Blood Count (CBC) on Day 1 (on Admission).

CBC	**Day 1**
**WBC (4.0-11.0 × 10**^3^ **/**µ**L)**	1.2 L
**RBC (3.8-5.3 × 10**^6^ **/**µ**L)**	1.22 L
**Hgb (11.5-15.3 g/**µ**L)**	4.6 L
**Hct (34.0%** to **45.0%)**	13.4 L
**MCV (80-100)**	110.3 H
**Platelets (155-400 × 10**^3^ **/**µ**L)**	13 L
**Neutrophil**s **(1.6-7.7 × 10**^3^ **/**µ**L)**	0.3 L
**Lymphocyte**s **(1.0-4.5 × 10**^3^ **/**µ**L)**	0.9 L
**Monocyte**s **(0.1-1.0 × 10**^3^ **/**µ**L)**	0.0 L

Abbreviations: WBC, white blood cell; RBC, red blood cell; Hgb, hemoglobin; Hct, hematocrit; MCV, mean corpuscular volume.

**Table 2.** Coagulation Panel, Factor VII Levels.

	Day 1	Day 3	Day 4	Day 10	Day 20	Day 23	Day 51^a^
Prothrombin time (12.5-14.2 s)	15.2 H	16.5 H		14.6 H		18.7 H	12.9
International normalized ratio (0.9-1.1)	1.22 H	1.36 H		1.16		1.61	0.98
Partial thromboplastin time (25.4-37.6 s)	25.2 L	28.8		21.6 L		37.3	
First cycle of chemotherapy begun			X				
Factor VII **(60**% to **150%**)						49 L	110
Mixing study					X		

a Forty days after completion of first cycle of chemotherapy.

Bone marrow aspirate on the second day of admission was performed and confirmed AML with cellularity of approximately 70% immature myeloid blasts, the phenotype of the neoplastic cells showed strong expression of CD117, and dim HLA-DR on flow cytometry. Cytogenetics revealed an abnormal karyotype of 47,x,t(x:8)(q13:q24.1),+8(6)/46,XX[14]. Twenty metaphases were analyzed and counted; 6 cells showed a reciprocal translocation involving the long arm of X chromosome and the long arm of chromosome 8, as well as a gain of 1 copy of chromosome 8. Molecular studies were positive for CEBPA, IDH1, U2FA1, JAK2, and negative for P53, FLT3, NPM1, and RUNX1.

The patient subsequently was treated with 7 days of daunorubicin (60 mg/m^2^/day) for 3 days and cytarabine 100 mg/m^2^/day (7 + 3 regimen) started on day 4 of her hospitalization. This regimen subsequently was administered for 2 cycles. However, 11 days after completing the first cycle, the patient’s PT increased to 18.7 with an INR of 1.61 despite PT being at 14.7 while receiving induction chemotherapy. Bone marrow aplasia secondary to induction chemotherapy with the 7 + 3 regimen was considered. However, in the setting of isolated and persistent PT prolongation more profound after induction chemotherapy, factor VII level deficiency was considered high in differentials. Furthermore, reviewing the literature showed clotting factor VII deficiency has been reported to be associated with trisomy chromosol abnormality involving chromosome 8. Correction of PT on mixing study on day 20 was suggestive of factor VII deficiency. Factor VII activity level was later obtained and confirmed to be low at 49% (normal level 60% to 150%). Forty days after completion of the first cycle and 2 weeks prior to starting the second cycle repeat factor VII activity level showed the factor VII levels had returned to normal level at 110%. In addition, her PT and INR were now within normal limits as well (Table 2).

Throughout her hospital stay, the patient continued to have intermittent mucocutaneous bleeding without any profound episodes of bleeding. She received multiple packed red blood cell transfusions to maintain hemoglobin >7 g/dL along as multiple platelet transfusion during her hospital stay. She did not require any fresh frozen plasma, factor concentrates, or any recombinant factor VII (RecFVIIa) products to control the bleeding. She was not diagnosed with any liver diseases, and her liver function tests remained within normal limits throughout.

The patient received her second cycle of chemotherapy approximately 2 months after her first cycle of induction chemotherapy. After completion of the second chemotherapy cycle, repeat bone marrow aspiration indicated no residual blasts and normalization of her hematologic abnormalities with normal white blood count, hemoglobin, platelet counts, and normal PT time. Flow cytometry demonstrated no residual blasts and repeat molecular and cytogenetic studies indicated the resolution of the previously detected abnormalities. She has remained in complete remission.

## Discussion

Acute myeloid leukemia is a clonal disease caused by genetic aberrations and both cytogenetic and molecular derangements are now frequently detected. Cytogenetic analyses in AML and acute lymphoblastic leukemia are a routine part of the initial evaluation of patients with leukemia.

Many nonrandom chromosome abnormalities are frequently identified with specific abnormalities conferring favorable, intermediate, or poor prognostic outcomes. In various instances, molecular studies of these abnormalities identify specific genes implicated in the process of leukemogenesis and are now used in combination with cytogenetics as diagnostic and prognostic markers, which help guide the clinicians in selecting the most effective therapies.^9^

This patient had chromosomal abnormalities including the presence of trisomy 8. Trisomy 8 (+8) is reported to be one of the more common numerical chromosome abnormalities in AML, with the occurrence of 9% of adult patients and classified as intermediate prognosis.^1^ Sole +8 AML is molecularly heterogeneous with mutations in RUNX1, ASXL1, IDH, DNMT3A, NPM1, and FLT3-ITD being the most frequent.^1^ The patient did have a detectable mutation in IDH1 specifically in addition to mutations in CEBPA and JAK-2 at a very low level.

There appears to be a link between trisomy 8 cytogenetic aberrations and the level or function of factor VII. Prior reports have highlighted the association of trisomy 8 and multiple hemorrhages and trisomy 8 controlling the expression of factor VII.^10,11^

It is not entirely clear why trisomy 8 leads to abnormalities of factor VII. However, since factor VII is a vitamin K–dependent factor, one possibility is the association of trisomy 8 with the NAD(P)H quinone pathway.

A high frequency of NAD(P)H: quinone oxidoreductase 1 (NQO1)C(609)T germline polymorphism in myelodysplastic syndrome/AML with trisomy 8 have been reported.^12^ NAD(P)H: quinone oxidoreductase 1 (NQO1) is an enzyme that in humans is encoded by the NQO1 gene. Typically thought of as a detoxification mechanism, NQO1 activity plays a role in vitamin K metabolism and is a well-documented component of pathways for mutagen and carcinogen activation. The NQO1 cytosolic enzyme catalyzes the 2-electron reduction of various quinones including vitamin K. This enzyme plays an important role in vitamin K metabolism. It reduces vitamin K to vitamin K hydroquinone, which will be utilized in the posttranslational γ-glutamyl carboxylation reactions. This reaction is essential for several proteins involved in blood coagulation cascade.^12,13^

The PT is the assay evaluating the extrinsic and common pathways of coagulation. PT measures the time, in seconds, for plasma to clot after adding thromboplastin.^14^ The speed of the extrinsic pathway is greatly affected by the level of functional factor VII. Factor VII is a vitamin K–dependent glycoprotein synthesized by the liver and has the shortest half-life in comparison to other coagulation proteins.

Factor VII deficiency could be inherited or acquired. Acquired factor VII deficiency can be diagnosed in the course of the exploration of a prolonged PT and is caused by vitamin K deficiency, severe liver disease, or use of vitamin K antagonist medications such as warfarin. It can present clinically as bleeding with an associated prolonged PT and low factor VII levels. This patient did not have a prior bleeding disorder but manifested clinically with mucocutaneous bleeding on presentation associated with slight but persistent elevation of PT and low factor VII level. However, the superficial bleeding, with slightly isolated prolonged PT, but severe thrombocytopenia, would suggest thrombocytopenia as a more prominent contributor to this coagulation defect.

Furthermore, the patient was not known to harbor any liver diseases; her liver function tests remained within normal limits even after induction chemotherapy with 7 + 3 regimen. She has never been on anticoagulants such as warfarin, any medication that would interfere with vitamin K metabolism, and never been on a vitamin K–deficient diet. Unfortunately, other vitamin K–dependent clotting factor levels such as factors II, IX, and X were not checked on this patient. This particular patient never had any signs or symptoms of disseminated intravascular coagulation to suggest possible low factor VII one would expect caused by accelerated consumption or catabolism phase. Thus, this documented isolated prolonged PT and the documented low factor VII levels were presumed to be acquired and strongly associated with her newly diagnosed acute leukemia.

While acquired factor VII deficiency has been previously reported in at least 31 cases, we can identify only 2 prior reported cases of acquired factor VII deficiency associated with AML described in the literature.^2,3^

In both of those 2 cases, the acquired factor VII deficiency was noted after induction chemotherapy and both after a documented *Aspergillus* infection.^3^ One was acquired factor VII deficiency secondary to the presence of factor VII inhibitor and the other case did not have a documented factor VII inhibitor only isolated low factor VII level. Both were complicated by bleeding. One of the cases had cytogenetic analysis and had a documented trisomy 8.^3^

Our patient had a documented trisomy 8 abnormality, an elevated PT, and a documented low factor VII level. These abnormalities corrected with systemic induction chemotherapy, which cleared the trisomy 8, normalized both the factor VII level and the PT. Even though hard to prove the direct link between all these abnormalities, here, we share the third case of AML with acquired factor VII deficiency and the second case of AML associated with trisomy 8 cytogenetic abnormality.

## Conclusion

This is an interesting case of concomitant acquired factor VII deficiency and trisomy 8 in association of newly diagnosed AML. Congenital deficiency of factor VII is well known but little is known about secondary or acquired factor VII deficiency. This case underscores the importance of a careful evaluation of a prolonged PT in patients presenting with leukemia. There are only 2 prior reported cases of acquired factor VII deficiency associated with AML described in the literature.^2,3^ To the best of our knowledge, this case is the third reported case of AML with acquired factor VII deficiency and the second reported case of AML with an associated acquired trisomy 8 cytogenetic abnormality.

It would be of interest for future studies to prospectively evaluate coagulation and to specifically investigate factor VII levels, factor VII inhibitors assays, document any associated fungal infections, and assess for NQO1 germline polymorphism in trisomy 8-related myelodysplastic syndrome/AML patients.
